# Repetitive N-WASP–Binding Elements of the Enterohemorrhagic *Escherichia coli* Effector EspF_U_ Synergistically Activate Actin Assembly

**DOI:** 10.1371/journal.ppat.1000191

**Published:** 2008-10-31

**Authors:** Kenneth G. Campellone, Hui-Chun Cheng, Douglas Robbins, Anosha D. Siripala, Emma J. McGhie, Richard D. Hayward, Matthew D. Welch, Michael K. Rosen, Vassilis Koronakis, John M. Leong

**Affiliations:** 1 Department of Molecular and Cell Biology, University of California Berkeley, Berkeley, California United States of America; 2 Department of Biochemistry and Howard Hughes Medical Institute, University of Texas Southwestern Medical Center, Dallas, Texas, United States of America; 3 Department of Molecular Genetics and Microbiology, University of Massachusetts Medical School, Worcester, Massachusetts, United States of America; 4 Department of Pathology, University of Cambridge, Cambridge, United Kingdom; Yale University School of Medicine, United States of America

## Abstract

Enterohemorrhagic *Escherichia coli* (EHEC) generate F-actin–rich adhesion pedestals by delivering effector proteins into mammalian cells. These effectors include the translocated receptor Tir, along with EspF_U_, a protein that associates indirectly with Tir and contains multiple peptide repeats that stimulate actin polymerization. In vitro, the EspF_U_ repeat region is capable of binding and activating recombinant derivatives of N-WASP, a host actin nucleation-promoting factor. In spite of the identification of these important bacterial and host factors, the underlying mechanisms of how EHEC so potently exploits the native actin assembly machinery have not been clearly defined. Here we show that Tir and EspF_U_ are sufficient for actin pedestal formation in cultured cells. Experimental clustering of Tir-EspF_U_ fusion proteins indicates that the central role of the cytoplasmic portion of Tir is to promote clustering of the repeat region of EspF_U_. Whereas clustering of a single EspF_U_ repeat is sufficient to bind N-WASP and generate pedestals on cultured cells, multi-repeat EspF_U_ derivatives promote actin assembly more efficiently. Moreover, the EspF_U_ repeats activate a protein complex containing N-WASP and the actin-binding protein WIP in a synergistic fashion in vitro, further suggesting that the repeats cooperate to stimulate actin polymerization in vivo. One explanation for repeat synergy is that simultaneous engagement of multiple N-WASP molecules can enhance its ability to interact with the actin nucleating Arp2/3 complex. These findings define the minimal set of bacterial effectors required for pedestal formation and the elements within those effectors that contribute to actin assembly via N-WASP-Arp2/3–mediated signaling pathways.

## Author Summary

Enterohemorrhagic *Escherichia coli* (EHEC) O157:H7 is a food-borne pathogen that causes diarrhea and life-threatening systemic illnesses. EHEC colonizes the intestine by adhering tightly to host cells and injecting bacterial molecules that trigger the formation of a “pedestal” below bound bacteria. These pedestals are generated by reorganizing the actin cytoskeleton into densely packed filaments beneath the plasma membrane. Pedestal formation is therefore not only important for EHEC disease, it provides a means to study how mammalian cells control their shape. We show here that two EHEC proteins, Tir and EspF_U_, are sufficient to trigger pedestal formation. Tir localizes to the mammalian plasma membrane, and its central function is to promote clustering of EspF_U_. EspF_U_ contains multiple repeat sequences that stimulate actin polymerization by binding N-WASP, a host protein that initiates actin assembly. Although a single repeat of EspF_U_ can generate pedestals, multi-repeat variants promote actin assembly cooperatively. One explanation for this synergy is that tandem repeats can potently trigger the formation of a complex of mammalian proteins that modulate the actin cytoskeleton. These findings define the minimal set of EHEC effectors required for pedestal formation and the elements within those effectors that confer their ability to alter cell shape.

## Introduction

Enterohemorrhagic *Escherichia coli* (EHEC) O157:H7 colonize the intestinal tract of cattle and other reservoir hosts without inducing disease, but cause severe diarrheal illness in humans that ingest contaminated materials [reviewed in 1]–[3]. The mode of epithelial colonization by EHEC reflects its membership in the attaching and effacing (AE) family of pathogens. These bacteria, which include enteropathogenic *E. coli* (EPEC) and *Citrobacter rodentium*, attach tightly to the intestinal epithelium, efface microvilli, and generate filamentous (F-)actin pedestals beneath sites of adherence. The formation of AE lesions is critical for pathogenesis, because mutations that abolish their biogenesis severely impair colonization [4]–[7]. Moreover, an EHEC mutant that forms AE lesions but possesses a diminished capacity to stimulate actin assembly is defective at expanding the initial infectious niche [8].

During infection, EHEC expresses a type III secretion system capable of translocating more than 30 effector proteins from the bacterium into the mammalian cell [9]. This delivery system is encoded by the locus of enterocyte effacement (LEE), which also contains several of the substrates for injection [reviewed in 10]–[11]. Among these LEE-encoded effectors is the translocated intimin receptor (Tir), which is essential for AE lesion formation. Tir is delivered into the host cell, where it localizes to the plasma membrane in a hairpin conformation that includes a central extracellular region that binds to intimin, a LEE-encoded adhesin. Intimin-Tir interaction promotes intimate attachment to the host cell, and also results in clustering of the N- and C-terminal cytoplasmic domains of Tir [12], which are capable of interacting with host proteins.

The Tir molecules from EHEC and EPEC both trigger actin assembly pathways that involve N-WASP, an actin nucleation-promoting factor [13]–[15]. N-WASP utilizes a C-terminal WH2/verprolin-connector-acidic (VCA) segment to activate the Arp2/3 complex, a major actin nucleator in cells [reviewed in 16]–[17]. Normally, N-WASP adopts an autoinhibited conformation in which its VCA domain is sequestered by an intramolecular interaction with a central GTPase binding domain (GBD). It can be activated by several stimuli, including Nck, an adaptor protein that binds to its proline-rich domain (PRD), and Cdc42, a small GTPase that binds the Cdc42-Rac binding (CRIB) sequence within the GBD [18]–[19]. When assayed using purified proteins in vitro, either Cdc42 or Nck is sufficient to stimulate N-WASP-Arp2/3–mediated actin assembly. However, under physiological conditions, N-WASP regulation is significantly more complex, since several proteins including WIP (WASP-interacting protein), bind to its N-terminal WH1 domain and influence its activation [20]. In fact, Cdc42 is insufficient to stimulate the native N-WASP/WIP complex [21].

EPEC pedestal formation involves activation of N-WASP by signaling initiated from the C-terminal cytoplasmic domain of its Tir protein, which is phosphorylated by host tyrosine kinases [22]–[24]. In fact, Tir is the only EPEC effector required for pedestal formation, since clustering of its ectopically expressed C-terminus in mammalian cells is sufficient to generate pedestals with high efficiency [25]. The dominant pathway for N-WASP stimulation by EPEC involves recruitment of Nck to one site of Tir tyrosine phosphorylation [reviewed in 26]–[27].

In contrast to EPEC, EHEC requires a second effector to trigger pedestal formation. This protein, EspF_U_ (also known as TccP), localizes beneath bound bacteria after delivery into host cells, co-precipitates with Tir, and promotes phosphotyrosine- and Nck-independent actin assembly [28]–[29]. Residues 456 to 458 in the cytoplasmic C-terminus of Tir are required for EspF_U_ recruitment and efficient pedestal formation [30]–[31], but because direct interactions between Tir and EspF_U_ have not been detected, additional factors are assumed to mediate their association.

EspF_U_ contains an N-terminal secretion signal followed by a C-terminus consisting of multiple nearly-identical 47-residue proline-rich peptide repeats [32]. EspF_U_ derivatives containing these repeats bind to a segment of N-WASP encompassing the GBD [27]–[28],[32]. Mutants containing the EspF_U_ N-terminus and as few as two repeats were shown to be capable of stimulating actin assembly using purified N-WASP and Arp2/3 in vitro and also could promote some degree of pedestal formation during infection of cultured cells, whereas derivatives containing only a single repeat did not [32].

These observations provide a framework for understanding the mechanisms by which EHEC triggers actin pedestal formation: Tir and EspF_U_ are central to actin assembly, the Tir C-terminus is critical for recruitment of EspF_U_ and other putative factors that may contribute to actin nucleation, and multiple proline-rich repeats of EspF_U_ are required for maximal signaling. However, important questions remain unanswered. For example, the potential roles for EHEC effectors other than Tir and EspF_U_ during actin assembly have not been defined, nor have the precise roles of the Tir C-terminus and/or the putative factors that mediate Tir-EspF_U_ interactions. In addition, whereas EspF_U_ can bind and activate purified recombinant N-WASP derivatives in vitro, it is unknown how accurately these assays might reflect N-WASP-stimulating activities in the context of the complex intracellular milieu, where other N-WASP binding partners like WIP likely modulate its autoinhibited state.

In the current study, we show that Tir and EspF_U_ are sufficient to trigger pedestal formation in the absence of any other EHEC factors. By analyzing EspF_U_ derivatives for the ability to bind native and recombinant N-WASP and stimulate N-WASP/WIP-mediated actin assembly in vitro and pedestal formation in cells, we arrive at a model in which the critical function of Tir is to promote clustering of the C-terminal repeats of EspF_U_. These repeats, in turn, activate N-WASP synergistically and lead to the formation of an Arp2/3-containing multi-protein complex that promotes robust actin polymerization.

## Results

### Intimin-mediated clustering of Tir and EspF_U_ is sufficient to promote actin pedestal formation

**Figure 1:**
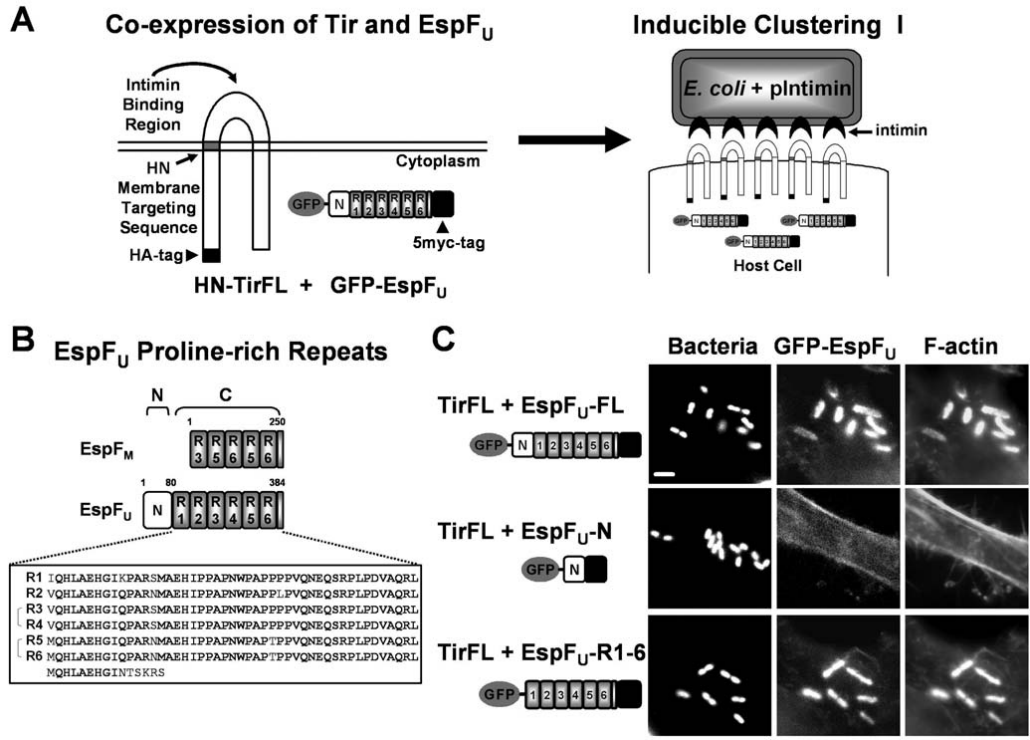
Clustering of Tir and EspF_U_ is sufficient to promote actin pedestal formation. (A) A full-length derivative of EHEC Tir (HN-TirFL) is depicted, featuring an HA-tag, N- and C-terminal cytoplasmic regions, an extracellular intimin-binding domain, and two transmembrane segments, including one derived from the Newcastle Disease Virus HN surface protein. A derivative of EspF_U_ tagged with GFP at its N-terminus and 5myc at its C-terminus (GFP-EspF_U_) is also shown. Treatment of transfected cells with a non-pathogenic strain of *E. coli* expressing EHEC intimin can promote clustering of membrane-localized HN-TirFL. (B) An alignment of EspF_U_ and the EHEC pseudogene EspF_M_ is shown, featuring an N-terminal EspF_U_ secretion signal and six nearly identical proline-rich peptide repeats plus one partial repeat at its C-terminus. The N-terminal boundary of the EspF_U_ repeats was assigned based upon alignment with the sequence of EspF_M_, which is missing the N-terminal translocation signal. (C) HeLa cells co-transfected with plasmids encoding HN-TirFL and GFP-EspF_U_ derivatives were treated with intimin-expressing *E. coli*, fixed, and stained with DAPI to identify bacteria and phalloidin to detect F-actin. All scalebars are 1 µm in length.

During infection, EHEC is capable of translocating more than 30 effector proteins into the host cell [9]. At least two of these effectors, EspF_U_ and EspF, are known to directly activate N-WASP [28]–[29],[33], while additional proteins stimulate signaling pathways that may also lead to N-WASP-mediated actin polymerization [34]. However, only two effectors, Tir and EspF_U_, have been shown to be crucial for EHEC pedestal formation in genetic deletion studies. Moreover, KC12, an EPEC strain in which EPEC *tir* has been replaced by EHEC *tir*, is defective at actin pedestal formation, whereas expression of EspF_U_ by KC12 allows pedestal formation at high efficiency in manner indistinguishable from that of EHEC [28]. These results are consistent with the possibility that Tir and EspF_U_ are the only effectors essential for EHEC-mediated actin pedestal formation. To definitively test whether EspF_U_ is the only effector in addition to Tir that is required for actin pedestal assembly, mammalian cells were transfected with plasmids encoding derivatives of EHEC Tir and EspF_U_ in the absence of all other bacterial factors. Tir was N-terminally tagged with an HA epitope, and its first transmembrane domain replaced with the transmembrane segment of the Newcastle Disease Virus HN surface protein to promote efficient plasma membrane localization [31]. EspF_U_ was fused to GFP at its N-terminus and a 5myc tag at its C-terminus to allow detection by fluorescence microscopy and immunoblotting, respectively (Figure 1A). In addition to full-length EspF_U_, we also generated GFP-fusions carrying only its N-terminal 88 residues (EspF_U_-N) or its C-terminal repeats (EspF_U_-R1-6) (Figure 1B). To cluster Tir in the plasma membrane, transfected cells were treated with a non-pathogenic strain of *E. coli* that was engineered to express intimin (Figure 1A), and binds selectively to Tir-expressing cells [31].

To evaluate actin pedestal formation on cells additionally co-expressing EspF_U_, only those cells exhibiting GFP fluorescence were examined. Adherent bacteria were identified by DAPI-staining and F-actin was visualized using fluorescent phalloidin. Bacteria that bound to cells co-expressing Tir and full-length EspF_U_ were associated with robust localized actin assembly, indicating that clustering of Tir in the presence of EspF_U_ is sufficient to trigger pedestal formation (Figure 1C). In addition, pedestals were formed on cells expressing the C-terminus of EspF_U_, but not cells expressing the N-terminus, indicating that the activity of EspF_U_ in pedestal formation resides entirely within the repeat region.

### Experimental clustering of a single EspF_U_ repeat bypasses the requirement for the Tir C-terminus during actin pedestal formation

**Figure 2:**
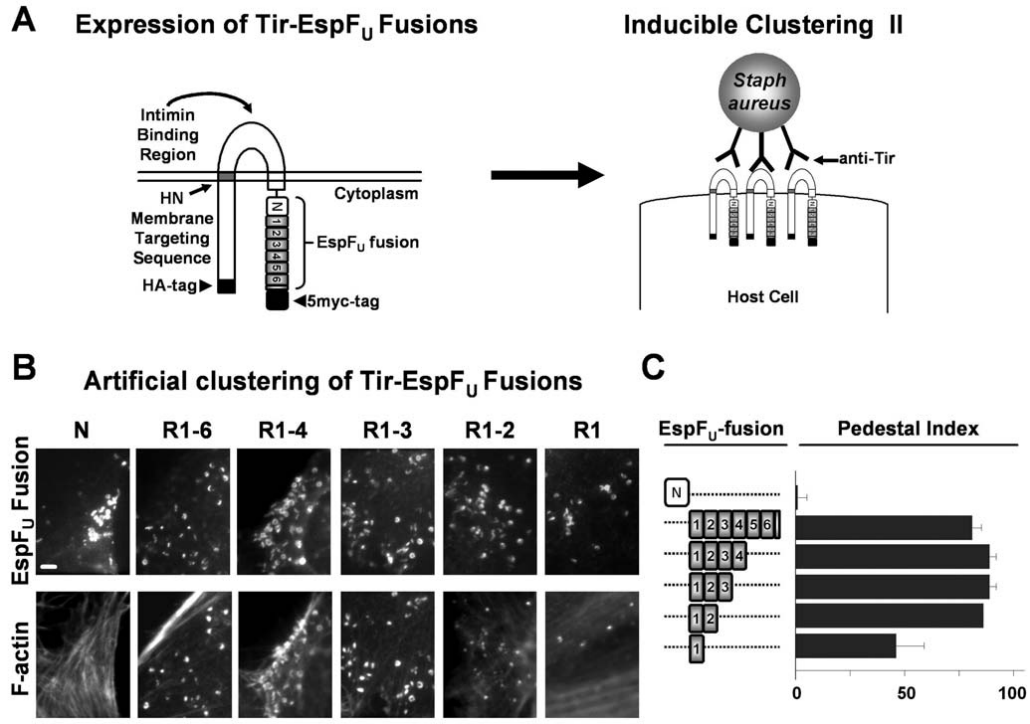
Experimental clustering of the EspF_U_ repeats bypasses the requirement for the Tir C-terminus during actin pedestal formation. (A) A fusion of membrane-targeted HN-TirFL to EspF_U_-myc is shown. Treatment of transfected cells with Tir antibodies and *S. aureus* particles can promote clustering of membrane-localized Tir-EspF_U_ fusions. (B) Murine fibroblast-like cells (FLCs) transfected with plasmids encoding Tir-EspF_U_ fusion constructs comprising the N-terminal domain or truncations of the C-terminal repeats were treated with Tir antibodies and *S. aureus*, fixed, and stained with HA antibodies to identify both transfected cells and *S. aureus* (which binds the fluorescent antibodies) and with phalloidin to detect F-actin. (C) Pedestal formation indices were determined by calculating the percentage of transfected cells harboring five or more *S. aureus* particles generating actin pedestals. Data represent the mean+/−SD from three experiments. The Tir-EspF_U_ R1 construct triggered actin assembly significantly less efficiently than the other truncations (p<0.05).

While deletion of the N-terminal cytoplasmic domain of Tir has a modest effect on actin assembly [31], the C-terminus of EHEC Tir contains a tripeptide sequence that is critical for both recruitment of EspF_U_ and pedestal formation [30]. Given that Tir and EspF_U_ do not appear to bind one another directly [28]–[32], and that EspF_U_ can activate N-WASP, the simplest model for pedestal formation is that the C-terminus of Tir serves to recruit a host factor that mediates the Tir-EspF_U_ interaction, thereby indirectly clustering EspF_U_ beneath the plasma membrane. If the major role of such a host protein is to act as an adaptor between Tir and EspF_U_, then artificial clustering of EspF_U_ at the plasma membrane should bypass the requirement for the C-terminus of Tir during pedestal formation. To test this possibility, we replaced the C-terminal cytoplasmic domain of HN-Tir with the C-terminal repeats of EspF_U_, expressed this Tir-EspF_U_ fusion in mammalian cells, and clustered it at the plasma membrane using antibodies directed against the extracellular region of Tir and formalin-fixed *Staphylococcus aureus* particles to engage Tir-bound antibodies (Figure 2A). We found that clustering of HN-Tir-EspF_U_-R1-6, which encompasses all 6 repeats, resulted in high levels of actin pedestal formation. Measurement of the fraction of cells harboring five or more pedestals yielded a pedestal “index” of nearly 90% (Figure 2B and 2C). As expected, the control HN-Tir-EspF_U_-N fusion did not trigger significant actin assembly.

The number of repeats present in the C-terminus of EspF_U_ from different EHEC isolates varies from two to eight, and an *espF_U_* mutant of the prototype strain EDL933 could not be complemented for pedestal formation by a plasmid encoding a truncation with only one repeat [32],[35]. To determine whether the quantity of repeats affects the efficiency of pedestal formation, we assessed actin assembly upon clustering of Tir-EspF_U_ chimeras containing variable numbers of EspFu repeats. All of the derivatives, including HN-Tir-EspF_U_-R1, a fusion containing only a single repeat, were capable of stimulating actin assembly (Figure 2B and 2C). Quantitation of the pedestal index revealed that HN-Tir-EspF_U_-R1 was roughly half as efficient at generating pedestals as the fusions containing multiple repeats (Figure 2C). These results suggest a remarkably simple model in which the central function of Tir is to promote clustering of the peptide repeats of EspF_U_ beneath the plasma membrane. In fact, clustering of a single repeat is sufficient to trigger actin pedestal formation.

### Multiple EspF_U_ repeats are required for full pedestal formation activity when expressed in the presence of full-length Tir

**Figure 3:**
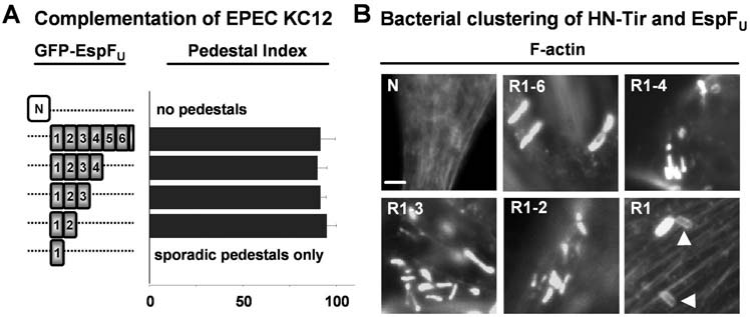
Multiple EspF_U_ repeats are required for full activity when expressed in the presence of full-length Tir. (A) FLCs transfected with plasmids encoding GFP-EspF_U_ truncations containing either the N-terminal domain or the C-terminal repeats were infected with EPEC KC12, fixed, and treated with DAPI to identify bacteria and phalloidin to detect F-actin. Pedestal indices were determined by calculating the % of GFP-EspF_U_-expressing cells harboring five or more actin pedestals. Data represent the mean+/−SD of three experiments. (B) HeLa cells co-transfected with plasmids encoding HN-TirFL and GFP-EspF_U_ truncations were infected with intimin-expressing *E. coli*, fixed, and treated with DAPI to identify bacteria and phalloidin to detect F-actin. Arrowheads indicate sites of less-intense F-actin assembly.

We next sought to further investigate the activity of EspF_U_ derivatives harboring varying numbers of repeats in experimental systems involving full-length Tir instead of Tir-EspF_U_ chimeras. To avoid potential variability in the efficiency of type III translocation of different EspF_U_ derivatives, we expressed GFP-EspF_U_ truncations in mammalian cells, and tested their ability to complement KC12, the equivalent of an EHECΔ*espF_U_* mutant [28], for actin pedestal formation. As expected [36], KC12 generated pedestals on cells expressing GFP-EspF_U_-R1-6, which carries the entire repeat region (Figure 3A). Moreover, all of the EspF_U_ derivatives that contained at least two repeats were also capable of complementing KC12 for pedestal formation. However, KC12 did not generate pedestals efficiently on GFP-EspF_U_-R1-expressing cells. Only 1–4 pedestals were occasionally observed on cells transfected with this variant (data not shown). This discrepancy in actin assembly cannot be attributed to differences in protein levels, because immunoblotting revealed that GFP-EspF_U_-R1 was well expressed in mammalian cells (data not shown).

To independently assess pedestal formation in the absence of other *E. coli* effectors that might compete with Tir or EspF_U_ for host target proteins, we also utilized the co-transfection approach described in Figure 1. HN-Tir along with GFP-EspF_U_ fusions carrying different numbers of repeats were expressed in mammalian cells and clustered by treating cells with intimin-expressing bacteria. When Tir was co-expressed with EspF_U_ constructs harboring multiple repeats, intensely staining F-actin pedestals were formed in high numbers (Figure 3B). On cells expressing HN-Tir and GFP-EspF_U_-R1, few bacteria were associated with intense pedestals, whereas most were associated with weakly staining F-actin (arrowheads). These results indicate that although EspF_U_ derivatives containing a single repeat can function for pedestal formation after clustering of full-length Tir in mammalian cells, the presence of 2–6 repeats is required for robust actin assembly.

### Multiple EspF_U_ repeats are required for efficient binding of native N-WASP in mammalian brain extract

**Figure 4:**
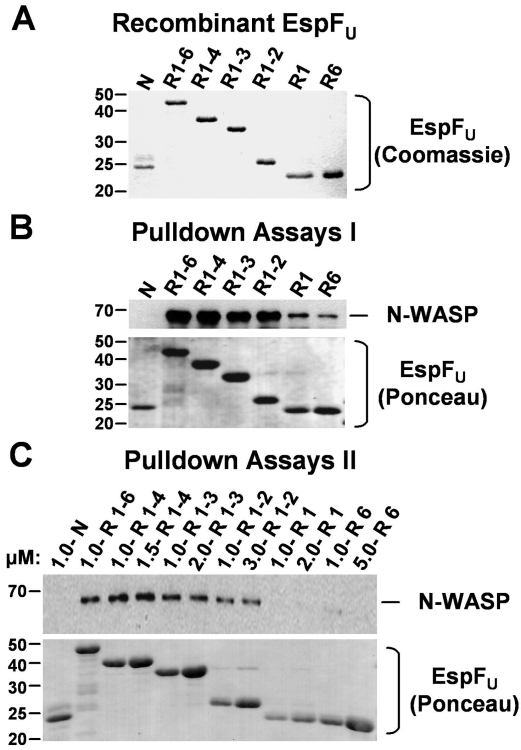
Multiple EspF_U_ repeats are required for efficient binding of N-WASP in brain extract. (A) N-terminally His10-tagged and C-terminally 5myc-tagged EspF_U_ derivatives were expressed in *E. coli*, purified, resolved by SDS-PAGE, and stained with Coomassie blue. (B) Cobalt-chelated magnetic particles were coated with saturating concentrations of EspF_U_ derivatives and subsequently incubated with porcine brain extract. The association of native N-WASP with EspF_U_-coated beads was assessed by SDS-PAGE followed by immunoblotting of bead eluates with antibodies to N-WASP and staining EspF_U_ with Ponceau S. (C) His-EspF_U_-myc constructs were added to brain extract at the indicated concentrations and collected using cobalt-chelated magnetic particles. The association of native N-WASP and EspF_U_ with the beads was assessed by immunoblotting of bead eluates with antibodies to N-WASP and staining EspF_U_ with Ponceau S.

Since actin pedestal formation varied depending on the number of EspF_U_ repeats, we next examined whether the efficiency of pedestal formation correlates with the ability of the different EspF_U_ truncations to interact with N-WASP. To characterize the interaction between N-WASP and derivatives of EspF_U_ harboring different numbers of repeats in a complex environment reflective of the mammalian cytosol, we generated protein extracts from pig brains, which are rich in actin cytoskeleton-associated factors, including N-WASP, WIP, and the Arp2/3 complex (data not shown). We also purified recombinant N-terminally His10- and C-terminally 5myc-tagged EspF_U_ derivatives consisting of 1 to 6 repeats, or as a negative control, an N-terminal fragment of EspF_U_ (Figure 4A). Magnetic beads saturated with these EspF_U_ truncations were then incubated with brain extracts, and N-WASP that bound to the beads was detected by immunoblotting. EspF_U_-R1-6, the derivative containing the entire C-terminus of EspF_U_, pulled down N-WASP, whereas the EspF_U_-N control fragment did not (Figure 4B). The efficiency of N-WASP pulldown appeared to be slightly lower for variants containing fewer than four repeats, whereas mutants containing only a single repeat bound N-WASP at substantially decreased levels (R1-6 = R1-4>R1-3 = R1-2≫R1 = R6).

Since it is possible that saturating beads with a single EspF_U_ repeat may artificially mimic derivatives with multiple repeats due to the density of protein clustered on the beads, we also assessed the interaction between EspF_U_ and native N-WASP under conditions where EspF_U_ concentrations could be more stringently controlled. Therefore, His-EspF_U_ derivatives were first added to extract at a defined concentration (1 µM), and then collected using cobalt-chelated magnetic particles. Immunoblotting revealed that N-WASP interacted efficiently with EspF_U_ derivatives containing 6 or 4 repeats (Figure 4C). Slightly lower levels of N-WASP bound to EspF_U_ proteins harboring 3 or 2 repeats, and constructs containing a single repeat unit pulled-down N-WASP at barely-detectable levels (R1-6 = R1-4>R1-3>R1-2>>>R1 = R6). Interestingly, increasing the concentration of EspF_U_ truncations had little effect on the quantity of N-WASP pulled-down by those proteins. Thus, although one repeat is capable of binding N-WASP in brain extracts, stepwise increases in binding are associated with the presence of additional EspF_U_ repeats within the same molecule, with an apparent plateau at 4–6 repeats.

### EspF_U_ repeats cause titratable increases in actin assembly in brain extract

**Figure 5:**
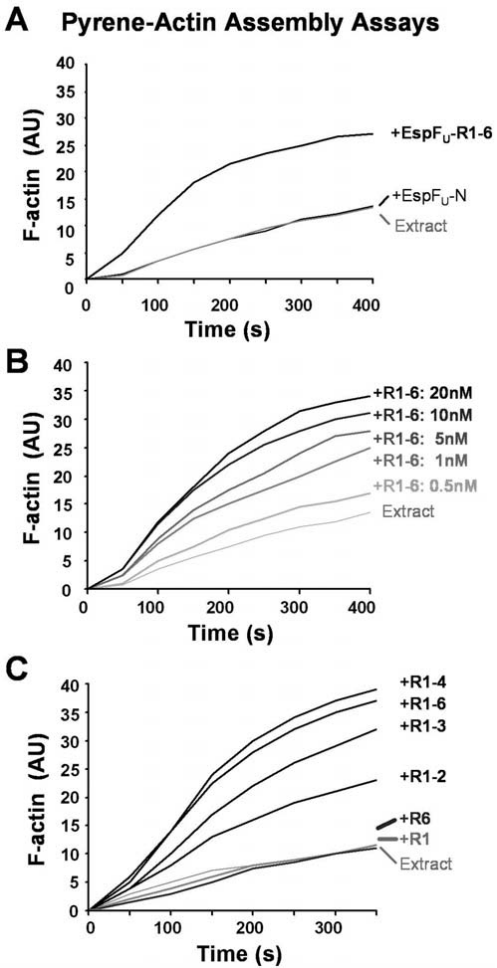
EspF_U_ repeats cause titratable increases in actin assembly in brain extract. (A) His-EspF_U_-myc constructs (20 nM each) were added to Arp2/3-enriched brain extract supplemented with 2.5 µM G-actin (10% pyrene labeled), and fluorescent actin polymerization, in arbitrary units (AU), was measured over time. EspF_U_ did not trigger actin assembly in the absence of extract (data not shown). (B) Pyrene-actin polymerization in the presence of brain extract and various concentrations of EspF_U_-R1-6 was measured over time. (C) Pyrene-actin polymerization in the presence of brain extract and EspF_U_ truncations containing different repeat segments (20 nM each) was measured over time.

EspF_U_ derivatives containing the N-terminal secretion domain and two or more repeats have been shown to stimulate N-WASP-Arp2/3–mediated actin assembly using purified proteins in vitro, while a truncation containing a single repeat barely triggered assembly [32]. To similarly assess the ability of EspF_U_ to promote actin polymerization, but in the presence of native components, we examined the ability of recombinant EspF_U_ variants to stimulate actin polymerization in brain extract. Purified EspF_U_ derivatives were added to extract supplemented with pyrene-labeled actin, and polymerization kinetics were measured fluorometrically [37]. Consistent with analyses of pedestal formation in cells, EspF_U_-R1-6 accelerated actin assembly in extract, while EspF_U_-N did not (Figure 5A). The degree of stimulation by EspF_U_-R1-6 was dose dependent, and peaked at 20 nM (Figure 5B), although polymerization rates were slower than those typically observed in a completely purified experimental system, presumably due to the presence of inhibitory factors in the extract. Concentrations above 20 nM did not increase the rate of assembly, and levels greater than 50 nM actually resulted in slower kinetics (not shown), implying that polymerization in extract is refractory in the presence of high levels of EspF_U_.

To characterize the relationship between the number of EspF_U_ repeats and the ability to stimulate actin assembly, pyrene-actin polymerization was also measured when the recombinant EspF_U_ truncations were added to extract. When tested at 20 nM, the optimal concentration for actin assembly mediated by the EspF_U_ derivative containing all 6 repeats, EspF_U_ variants containing 2, 3, or 4 repeats accelerated actin assembly, and the rate of polymerization positively correlated with the number of repeats that were present (Figure 5C). In contrast, neither EspF_U_-R1 nor EspF_U_-R6, the truncations containing a single repeat, caused a significant acceleration of actin assembly in this assay system. Notably, the kinetics of actin assembly in these studies correlated with the ability of the EspF_U_ constructs to interact with N-WASP in the extract (Figure 4). Consistent with such results, increasing the concentration of EspF_U_ variants harboring less than 3 repeats did not enhance actin assembly (data not shown). Collectively, these data indicate that the presence of multiple repeats within the same polypeptide is important for triggering actin polymerization in an extract system designed to mimic a complex native environment.

### EspF_U_ repeats synergistically activate N-WASP/WIP–mediated actin assembly in vitro

**Figure 6:**
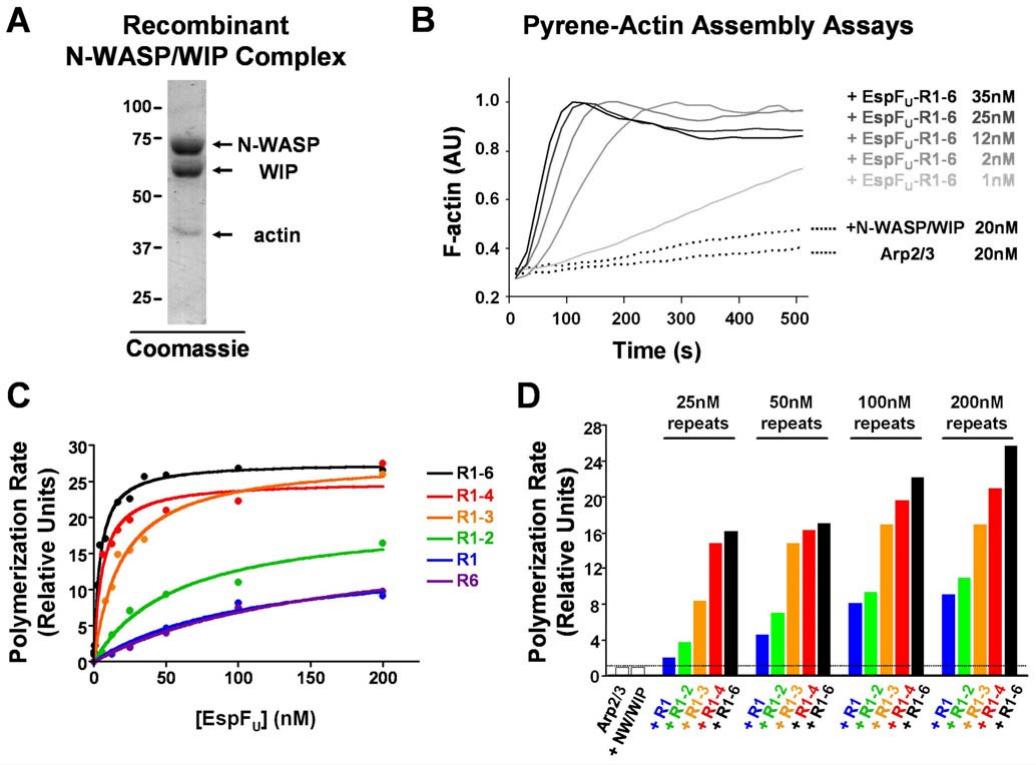
EspF_U_ repeats synergistically activate actin assembly mediated by recombinant N-WASP/WIP complex in vitro. (A) N-terminally His6-Flag-tagged N-WASP and His6-Myc-tagged WIP were co-expressed in insect cells, purified as a stoichiometric complex, resolved by SDS-PAGE, and stained with Coomassie blue. (B) Actin (2 µM) was polymerized in the presence of Arp2/3 complex, N-WASP/WIP complex, and the indicated concentrations of EspF_U_. F-actin fluorescence was measured in arbitrary units (AU). (C) Actin polymerization was examined in the presence of 20 nM Arp2/3 complex, 20 nM N-WASP/WIP complex, and the indicated concentrations of EspF_U_ derivatives. Polymerization rates at half-maximal F-actin concentrations were measured relative to the rate of polymerization in control Arp2/3+N-WASP/WIP samples lacking EspF_U_. Curves were fit using Prism software. (D) Actin polymerization was measured as in (C), except that EspF_U_ concentrations have been scaled to the number of repeats in each protein.

Under physiological conditions, N-WASP stably associates with WIP or it homologs CR16 and WIRE/WICH [21],[38]. In addition, WIP is capable of inhibiting Cdc42-mediated activation of N-WASP [20] and, unlike recombinant N-WASP, the native N-WASP/WIP complex is insensitive to Cdc42 treatment [21], suggesting that regulation of N-WASP in isolation may not accurately reflect physiological N-WASP regulation in vivo. We therefore examined whether EspF_U_ can trigger Arp2/3-mediated actin assembly in the presence of an N-WASP/WIP complex. We purified a recombinant complex containing N-WASP and WIP at 1∶1 stoichiometry and also contained a trace amount of insect cell actin (see Materials and Methods; Figure 6A). We also purified recombinant Arp2/3 complex [39], and examined the basal activity of the N-WASP/WIP complex using the pyrene-actin polymerization assay. Consistent with the predicted autoregulation of N-WASP, the recombinant N-WASP/WIP complex caused only a small increase in actin assembly kinetics when compared to Arp2/3 alone (Figure 6B).

To determine if EspF_U_ could trigger actin assembly in this purified system, pyrene-actin polymerization was measured in the presence of recombinant EspF_U_-R1-6, N-WASP/WIP, and Arp2/3 complex. Consistent with analyses of pedestal formation in cells and actin assembly in extracts, EspF_U_-R1-6 accelerated actin assembly in this reconstituted system (Figure 6B). In the presence of 20 nM N-WASP/WIP and 20 nM Arp2/3, activation appeared to saturate at approximately 35 nM EspF_U_-R1-6.

We next examined the abilities of the EspF_U_ truncations to stimulate actin assembly. By identifying the length of time each reaction took to reach half of the maximal F-actin concentration and measuring the rates of actin polymerization at these times, we directly compared the activity of each construct (Figure 6C). These experiments revealed that EspF_U_-R1-6 and EspF_U_-R1-4 were nearly indistinguishable at stimulating actin assembly, as the concentrations of these proteins that were required for reaching half of the maximal polymerization rate were approximately 4.0 nM and 5.7 nM, respectively. EspF_U_-R1-3 was less active than either the 6- or 4-repeat constructs at all concentrations tested, and it stimulated half-maximal assembly at a concentration of 19.4 nM. EspF_U_-R1-2 was substantially less active than the 3-repeat derivative (half-maximal at 78 nM), while the single repeat constructs EspF_U_-R1 and EspF_U_-R6 were even less active (half-maximal at 99–117 nM). Even when present at very high concentrations (>500 nM), the EspF_U_ derivatives harboring 1–2 repeats could not accelerate polymerization to rates comparable to those elicited by 35 nM EspF_U_-R1-6 (data not shown). Thus, in the presence of purified N-WASP/WIP and Arp2/3, the number of EspF_U_ repeats positively correlates with the rate of actin assembly.

To compare the activity of the EspF_U_ derivatives on the basis of repeat numbers, we normalized protein concentrations to the quantity of repeat units within each polypeptide. For example, 4.17 nM of the 6-repeat construct was considered equivalent to 8.33 nM of a 3-repeat truncation and 25 nM of a single repeat. Scaling of the data in this manner revealed that EspF_U_ derivatives containing greater numbers of repeats within the same protein had substantially higher activity than smaller constructs (Figure 6D). For example, when protein concentrations were normalized to 25 nM of repeats, the 6-repeat protein was actually twice as active as the 3-repeat protein, which was more than twice as active as the 2-repeat protein, which was roughly twice as active a 1-repeat protein. A similar trend was apparent at higher concentrations (50–200 nM), as longer EspF_U_ constructs always had greater levels of activity than smaller derivatives containing equimolar amounts of repeats. Hence, EspF_U_ repeats cooperate when present within the same protein to promote synergistic activation of N-WASP/WIP and Arp2/3-mediated actin assembly.

### The GBD of N-WASP is a dominant negative inhibitor of EHEC pedestal formation and binds with high efficiency to a single EspF_U_ repeat in yeast two-hybrid assays

**Figure 7:**
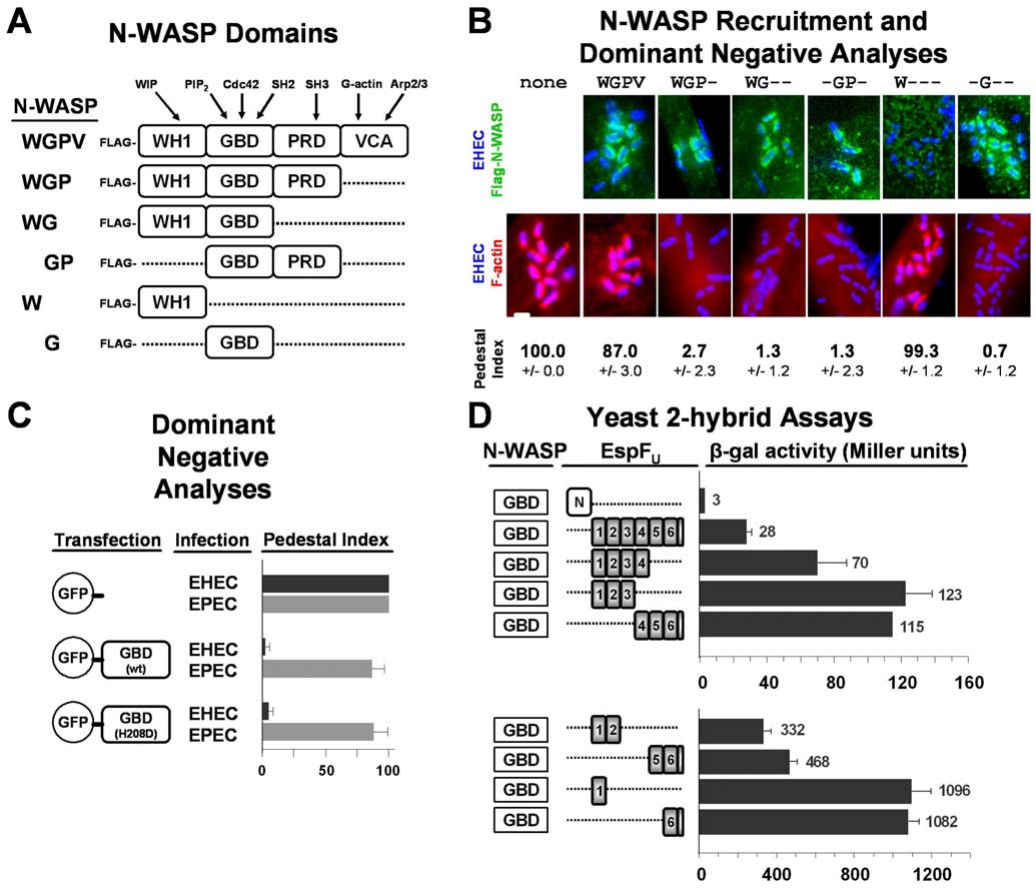
The GTPase-binding domain (GBD) of N-WASP is a dominant negative inhibitor of EHEC pedestal formation and binds with high efficiency to a single EspF_U_ repeat in yeast two-hybrid assays. (A) The modular structure of N-WASP is depicted, featuring WASP homology-1 (WH1), GTPase-binding (GBD), proline-rich (PRD), and WH2/verprolin-connector-acidic (VCA) domains. Several N-WASP-binding partners are shown above their interacting domains. (B) HeLa cells transfected with plasmids encoding Flag-N-WASP constructs were infected with EHECΔ*dam* (a mutant that binds to mammalian cells and generates pedestals with considerably higher efficiency than wild type EHEC [44]), fixed, and treated with DAPI to identify bacteria, a Flag antibody to visualize tagged N-WASP (top panels), and phalloidin to detect F-actin (bottom panels). Flag-N-WASP recruitment was only evaluated in cells expressing low levels of these tagged proteins (top panels), while effects on actin pedestal formation were only assessed in cells expressing high levels of Flag-N-WASP (bottom panels). Pedestal formation indices were determined by calculating the percentage of mock-transfected or Flag-N-WASP overexpressing cells harboring five or more actin pedestals. Data represent the mean+/−SD of three experiments. (C) HeLa cells expressing GFP alone, a GFP-tagged GBD, or a GFP-tagged GBD H208D point mutant were infected with EHECΔ*dam* or EPEC and treated with DAPI to identify bacteria and phalloidin to detect F-actin. Pedestal formation indices were determined as in (B). (D) Plasmids encoding the N-WASP GBD fused to the LexA DNA-binding domain and EspF_U_ fragments fused to the Gal4 transcriptional activation domain were co-transformed into a yeast two-hybrid reporter strain. Data represent the mean+/−SD of β-galactosidase activity for three co-transformants for each pairwise combination.

Insight into the ability of multi-repeat EspF_U_ derivatives to pulldown N-WASP from brain extracts and stimulate N-WASP/WIP-mediated actin assembly in vitro would be enhanced by a better understanding of EspF_U_ binding by N-WASP, which is mediated by the GBD [28]–[29]. To further test the functional significance of this interaction during pedestal formation, we transfected mammalian cells with constructs expressing Flag-tagged N-WASP variants, each encompassing a different combination of N-WASP domains (Figure 7A), and then examined pedestal formation upon infection with EHEC. Consistent with previous observations showing that N-WASP residues 226–274 within the GBD mediate recruitment of N-WASP to sites of EHEC attachment [14], all tagged N-WASP derivatives containing the GBD (residues 151–273) were recruited to sites of bacterial adherence (Figure 7B, top row). In addition, quantitation of pedestal formation on transfected cells revealed that neither the N-terminal WH1 domain nor full-length N-WASP had a substantial effect on actin polymerization, whereas overexpression of each of the 4 GBD-containing derivatives that lacked the VCA domain, which is necessary for Arp2/3 activation, effectively abolished pedestal formation (Figure 7B, bottom row). This inhibition was specific, because overexpression of the GBD did not affect actin assembly triggered by an EPEC strain that generates pedestals independently of EspF_U_ (Figure 7C). As predicted by mapping of requirements for N-WASP recruitment to EHEC [14], an H208D point mutation that abrogates N-WASP binding by the GTPase Cdc42 had no effect on the inhibitory activity of the GBD. These results indicate that the interaction between EspF_U_ and the C-terminal portion of the N-WASP GBD is important for pedestal formation in mammalian cells.

Next, to begin to assess whether the positive correlation between the number of repeats within an EspF_U_ derivative and its ability to activate actin assembly is reflected in binding to the N-WASP GBD, we examined this interaction in yeast two-hybrid assays. As demonstrated previously [28], the N-WASP GBD interacted with the C-terminus of EspF_U_ that contains the repeat sequences, but not the EspF_U_ N-terminus (Figure 7D). In addition, all of the C-terminal subfragments of EspF_U_, even those containing only a single repeat, interacted with the GBD in these assays. However, the degree of interaction did not positively correlate with increasing numbers of repeats. Rather, the number of repeats in EspF_U_ inversely correlated with the degree of expression of the *lacZ* reporter. Although reporter activity in the yeast two-hybrid assay reflects a number of parameters, including levels of expression and nuclear import of the binding partners, these results provide no evidence that the more efficient activation by multi-repeat EspF_U_ derivatives is a consequence of cooperativity in GBD binding.

### Increasing the number of EspF_U_ repeats does not alter affinity for the GBD, but promotes the formation of an Arp2/3-containing complex

**Figure 8:**
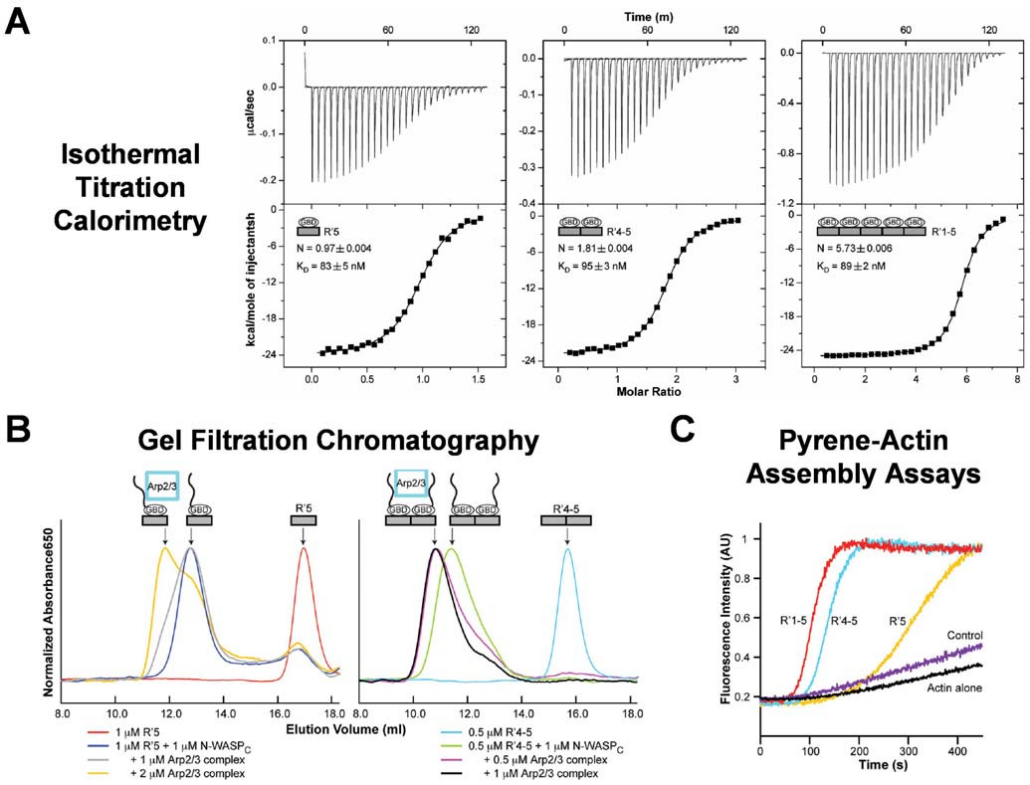
Increasing the number of EspF_U_ repeats does not alter affinity for the GBD, but promotes the formation of an Arp2/3-containing complex. (A) Isothermal titration calorimetry analyses of the interactions between WASP GBD and EspF_U_ fragments are shown. The GBD was titrated into R′5 (left), R′4-5 (middle), or R′1-5 (right). Raw and integrated heats of injections are shown in upper and lower panels, respectively. Black lines in the lower panels show fits of data into a single-affinity, multi-site binding model. Fits of the data for R′4-5 and R′1-5 to models with two different affinities were not statistically improved over the single-affinity model. (B) Interactions between Arp2/3 complex and N-WASP_C_ in complex with Alexa647-labeled EspF_U_ R′5 or R′4-5 were examined by gel filtration chromatography. The A_650_ profile is shown. (C) Actin (4 µM) was polymerized by itself (black curve), or in the presence of 10 nM Arp2/3 complex plus either 0.5 µM WASP GBD-VCA (purple, control), 0.5 µM GBD-VCA+1 µM R′5 (yellow), or 0.5 µM GBD-VCA+0.5 µM R′4-5 (blue), or 0.5 µM GBD-VCA+0.2 µM R′1-5 (red). F-actin fluorescence was measured in arbitrary units (AU). Note that R′5, R′4-5, and R′1-5 were used at the same total repeat concentration.

To further assess potential cooperativity in GBD binding by EspF_U_, we examined the interactions of EspF_U_ derivatives with the GBD of WASP, the hematopoetic-specific homologue of N-WASP that has been shown to also promote actin pedestal formation in cells [14]. Recombinant EspF_U_ proteins containing 5, 2, or 1 repeats (R′1-5, R′4-5, and R′5, respectively) were generated with N- and C-terminal repeat boundaries based on recent structural studies that defined the GBD-binding sequences of EspF_U_ [40]–[42]. The affinity of the WASP GBD for these EspF_U_ constructs was determined using isothermal titration calorimetry and yielded predicted differences in stoichiometry (i.e., 5.7, 1.8, and 1.0 for the 5, 2, and 1 repeat derivatives, respectively) but similar dissociation constants (i.e., 83 nM, 95 nM, and 89 nM, respectively) (Figure 8A). Thus, variation in the number of EspF_U_ repeats is not associated with differences in affinity for the GBD.

We next tested whether oligomerization of N-WASP by adjacent EspF_U_ repeats instead might promote binding of the Arp2/3 complex. N-WASP_C_, a C-terminal fragment of N-WASP that contains the GBD, PRD, and VCA domain, is a fragment previously shown to function in pedestal formation [14]. To determine whether EspF_U_ derivatives differing in repeat number also differed in their ability to form a complex containing N-WASP_C_ and Arp2/3, EspF_U_ variants containing 1 or 2 repeats, fluorescently labeled at their N-termini with AlexaFluor647, were incubated with equivalent molar concentrations of N-WASP_C_ in the absence or presence of the Arp2/3 complex. The relative size of EspF_U_-containing complexes, detected by absorbance at 650 nM, was then determined by gel filtration chromatography. The single EspF_U_ repeat bound N-WASP at essentially stoichiometric levels when mixed in equimolar (2 µM) quantities, as determined by an increase in the apparent size (i.e., earlier elution profile) of EspF_U_ (Figure 8B left, red vs. blue peaks). The addition of 2 µM (i.e., a two-fold higher molar concentration) Arp2/3 caused ∼60% of the EspF_U_ to shift to an even more rapidly eluting fraction indicative of an EspF_U_-N-WASP-Arp2/3 complex (Figure 8B, yellow profile). Very little of this complex was detected using 1 µM Arp2/3 (Figure 8B left, small shoulder in gray profile).

When 1 µM N-WASP_C_ was incubated with 0.5 µM of the 2-repeat EspF_U_ derivative (i.e., containing a normalized repeat concentration), the two proteins bound one another at essentially stoichiometric levels (Figure 8B, right, compare light blue and green profiles). However, in contrast to the requirement for 2 µM Arp2/3 to observe appreciable complex formation with a single repeat, the addition of as little as 0.5 µM Arp2/3 to N-WASP_C_ and the two repeat derivative resulted in the formation of an Arp2/3-containing complex (Figure 8B right, blue or pink profiles, respectively). The greater complex formation even at lower Arp2/3 concentration indicates that the ability of tandem EspF_U_ repeats to assemble N-WASP dimers (or by implication higher order multimers) may facilitate binding of the Arp2/3 complex, providing a likely source of inter-repeat cooperativity.

Since the assay demonstrating inter-repeat cooperation of EspF_U_ for the formation of an Arp2/3-containing complex described above involved the addition of only EspF_U_, N-WASP_C_, and Arp2/3, N-WASP-associated proteins such as WIP should not be required for cooperativity between the EspF_U_ repeats in actin assembly. Furthermore, domains of WASP (or N-WASP) other than the GBD (which binds EspF_U_) and VCA (which binds Arp2/3) should also be dispensable. We therefore tested whether the EspF_U_ repeats were capable of synergistically activating a minimized (and normally autoinhibited [40]) derivative of WASP containing only the GBD and VCA domain. Similar to results using EspF_U_ truncations and an N-WASP/WIP complex, pyrene actin assays using WASP GBD-VCA revealed that the number of EspF_U_ repeats correlated with increased actin polymerization rates, even when protein concentrations were normalized to repeat units (Figure 8C). These results indicate that synergy in actin assembly require no WASP sequences other than the GBD and VCA domain.

## Discussion

In order to better understand EHEC pedestal formation, we first sought to define the minimal set of bacterial effectors essential to this process. We found that EspF_U_ is likely the only EHEC effector besides Tir that is required for pedestal formation, because pedestals can be induced on mammalian cells that express these two factors in the absence of any other bacterial proteins. In addition, the cytoplasmic C-terminus of Tir could be functionally replaced with the C-terminus of EspF_U_, indicating that the only critical function of this Tir domain is to recruit the EspF_U_ repeats to sites of EHEC attachment. The C-terminus of the prototype EspF_U_ protein consists of six 47-residue proline-rich repeats, and a fusion containing a single repeat unit retained significant ability to generate pedestals when clustered at the plasma membrane. This is consistent with a recent report describing actin recruitment to the plasma membrane after artificially clustering a single repeat with antibodies [42]. That study, as well as the recent report of the structure of a single EspF_U_ repeat bound to the WASP GBD [41], revealed that EspF_U_ binds to the autoinhibitory region within the GBD that normally interacts with the VCA domain, a finding consistent with previous mapping studies [14],[27],[32]. Therefore, the bare minimum components required for pedestal formation are the domains of Tir that facilitate its membrane localization and clustering by intimin, and a single GBD-binding EspF_U_ peptide repeat.

Within the Tir C-terminal domain, a tripeptide sequence (NPY_458_) is required for recruitment of EspF_U_ and actin pedestal formation [30], but a direct interaction between Tir and EspF_U_ has not been detected, suggesting that they interact indirectly. The delineation of Tir and EspF_U_ as the only bacterial effectors required for pedestal formation indicates that the putative adaptor that mediates this interaction is of host origin. Efficient association of EspF_U_ with the putative adaptor linking it to Tir may require multiple EspF_U_ repeats, because when Tir and EspF_U_ were expressed separately rather than as a single fusion protein, at least two repeats were required for robust EspF_U_ function. Consistent with these results, a translocated 2-repeat EspF_U_ derivative, but not a 1-repeat derivative, localized to sites of bacterial attachment [32].

Aside from promoting Tir-EspF_U_ interactions, this host factor may itself promote some level of actin assembly, because residual pedestals can form at low levels in the absence of EspF_U_ [28]. Cortactin, which has the ability to stimulate Arp2/3 and contributes to pedestal formation via an unknown mechanism, can interact with both Tir and EspF_U_, but binds the Tir N-terminus [36], a domain that is largely dispensable for actin assembly [31]. Regardless of the identity of the adaptor, the finding that a hybrid protein containing Tir fused directly to the EspF_U_ repeats is fully functional for pedestal formation indicates that neither the adaptor nor the C-terminus of Tir play any essential role in actin assembly other than to recruit EspF_U_. Thus, EspF_U_ is the primary effector that signals to the actin cytoskeleton during pedestal formation.

In addition to promoting more efficient recruitment to Tir, the presence of multiple repeat units in EspF_U_ provides more robust signaling function, because a Tir-EspF_U_ fusion carrying two or more repeats triggered pedestal formation at levels 2-fold higher than a fusion carrying a single repeat. Recently, a full-length repeat region was shown to recruit GFP-actin to the plasma membrane somewhat (∼20%) more frequently than a single repeat [42]. That report and others have also shown that multi-repeat EspF_U_ derivatives promote more efficient actin assembly in vitro than a single-repeat derivative [32],[42]. Such studies have relied upon analyses of recombinant N-WASP or small N-WASP fragments containing the minimal autoinhibitory module, a GBD-VCA fusion. However, in recent years it has become apparent that in the cell cytoplasm the intrinsic activity of N-WASP may be significantly modulated through interactions with other factors [20]–[21]. We therefore examined EspF_U_-induced actin polymerization in brain extracts, and found that in this complex environment, the number of repeats correlated both with the ability of EspF_U_ derivatives to interact with native N-WASP and to stimulate actin assembly. Single-repeat constructs did not accelerate polymerization in these assays, possibly due to competition with endogenous N-WASP activators or the presence of inhibitory factors in the extract.

Under physiological conditions, N-WASP is stably associated with WIP, a protein that binds to its N-terminal WH1 domain and influences its activation [20]–[21]. We found that EspF_U_ constructs are capable of potently activating Arp2/3-mediated actin assembly in the presence of a recombinant N-WASP/WIP complex, and as predicted based upon N-WASP pulldown assays and measurements of actin polymerization in extracts, EspF_U_ derivatives containing greater numbers of repeats stimulate actin assembly more rapidly. Interestingly, when we normalized the EspF_U_ derivatives to the concentration of repeats and quantitated actin assembly rates, we found that increasing the number of repeats within individual proteins does not simply result in additive increases in actin polymerization. Instead, the presence of multiple repeats in the same derivative enhances N-WASP/WIP-mediated actin assembly synergistically.

It is possible that this enhancement phenotype is due to cooperativity among the repeats in EspF_U_-N-WASP binding. Indeed, an earlier study reported that the dissociation constants (Kd) for the EspF_U_-N-WASP interaction varied depending on the numbers of repeats (e.g., 3.6 nM for 6 repeats, 6.4 nM for 4 repeats, and 11 nM for 2 repeats, and no measurable binding for 1 repeat) [32]. However, these values were calculated on the basis of in vitro actin assembly assays that involve the formation of an N-WASP-Arp2/3 complex, and may not simply reflect the interaction between EspF_U_ and N-WASP. Similarly, the more efficient pulldown of N-WASP by multi-repeat EspF_U_ derivatives in brain extracts that we observed could be influenced by other endogenous factors. Although a previous study found that 2 repeats is the minimal N-WASP-binding module within EspF_U_ in gel overlay assays [32], we found that a single repeat bound to the GBD with high efficiency in yeast two-hybrid assays. Furthermore, the Kd for binding of purified 1-, 2-, or 5-repeat EspF_U_ derivatives to the WASP GBD were all similar to one another (i.e., 83–95 nM), and within an order of magnitude of that measured for the N-WASP GBD (18 nM; [42]). These results provide convincing evidence that the EspF_U_ repeats do not bind to the GBD in a cooperative fashion.

An alternative explanation for inter-repeat cooperativity during actin polymerization is that an EspF_U_-N-WASP-Arp2/3 complex can be formed more efficiently. The major threshold for pedestal-forming function apparently resides within 2 EspF_U_ repeats, and we were unable to detect substantial differences in the frequency or intensity of actin pedestal formation promoted by EspF_U_ proteins harboring 2, 3, 4, or 6 repeats (although in vitro actin assembly assays indicate that the number of repeats present in an EspF_U_ derivative correlates positively with function). Similarly, analysis of a diverse collection of *espF_U_*-containing *E. coli* strains showed that all EspF_U_ proteins contain at least 2 repeats [35]. Therefore, we compared the ability of EspF_U_ derivatives containing 1 versus 2 repeats to enter into a complex with recombinant N-WASP and Arp2/3 complex. As predicted from the affinities of EspF_U_ fragments for the GBD, the single and two repeat derivatives bound to N-WASP indistinguishably. However, the 2-repeat protein formed a complex with both N-WASP and Arp2/3 with much greater efficiency. These experiments suggest that N-WASP multimers assembled by tandem EspF_U_ repeats may have higher affinity for Arp2/3 complex. As predicted from such a model, the inter-repeat cooperativity was revealed during in vitro actin assembly assays using a WASP derivative containing only the EspF_U_-binding GBD and the Arp2/3-binding VCA domain. Further study will be required to illuminate the molecular basis of this enhanced interaction.

Interestingly, the multivalency that is clearly a critical property of EspF_U_ is likely an important feature of other Arp2/3-mediated processes that are triggered by microbial pathogens. Pedestal formation by EPEC, which occurs in an EspF_U_-independent manner, requires clustering of Tir, a step that may mimic multivalent binding. The critical EPEC Tir phosphopeptide has no activity when added in soluble form in standard pyrene actin assays [S. Rankin, unpub. obs.], but potently stimulates actin assembly when clustered on a bead [25]. EspF, an EHEC effector that is 35% similar to EspF_U_ but not involved in pedestal formation [28], also uses peptide repeats to activate N-WASP in vitro [33]. Finally, the *Shigella* IcsA (VirG) protein uses repetitive sequences to bind N-WASP and promote actin-based intracellular motility [43]. Future investigation into how the multivalent nature of EspF_U_ promotes actin assembly is likely to provide insight into related phenomena central to multiple pathogenic processes.

## Materials and Methods

### Bacteria, plasmids, and cell lines

The EHEC *dam* mutant used in this study was derived from TUV93-0, a Shiga toxin-deficient version of the prototype O157:H7 strain EDL933 [44]. The EPEC bacterium was the prototype O127:H6 strain, JPN15/pMAR7. EPEC KC12, which contains the EPEC *tir-cesT-eae* operon replaced with the corresponding EHEC operon, has also been described [45]. A non-pathogenic strain of *E. coli* (MC1061) expressing EHEC intimin was also described elsewhere [31]. For yeast two-hybrid analyses, *espF_U_* derivatives were generated by PCR from EDL933 genomic DNA and cloned into the EcoRI and BamHI sites of pGAD424 [28] to create fusions between an N-terminal GAL4AD and C-terminal 5myc tag. For transfections, *espF_U_* fragments were subcloned into the KpnI and BamHI sites of pKC425 [46] to generate fusions between an N-terminal GFP-tag and C-terminal 5myc tag. For protein expression and purification, most *espF_U_* derivatives were subcloned into the NdeI and XbaI sites of the vector pET16b (Novagen) to create fusions between an N-terminal His10-tag and C-terminal 5myc tag. Some EspF_U_ constructs contained only an N-terminal His-tag (Figure 8) [41]. For Tir-EspF_U_ hybrids, *espF_U_-myc* derivatives were subcloned into the KpnI and XbaI sites of the HN-Tir-related vector pKC689 [31]. EspF_U_ truncations contained amino acids 1-88 (N), 80-384 (R1-6), 80-275 (R1-4), 80-228 (R1-3), 80-181 (R1-2), 80-134 (R1), 229-384 (R4-6), 276-384 (R5-6), 323-384 (R6), 80-314 (R′1-5), 221-314 (R′4-5), and 268-314 (R′5). For dominant negative transfections, rat N-WASP derivatives were generated by PCR with an N-terminal Flag-tag using domain boundaries described previously [28] and cloned into the KpnI-EcoRI sites of the vector pCDNA3 (Invitrogen). N-terminally GFP-tagged derivatives of the GBD were generated by subcloning into pKC425. Plasmids for expression of membrane-targeted HN-TirFL in mammalian cells and expression of the N-WASP GBD fused to the LexA DBD in pBTM116 have been described previously [28],[31]. Plasmids for expression of His-Flag-N-WASP and His-Myc-WIP in insect cells are described elsewhere (ADS and MDW, submitted). N-terminally His- or GST-tagged WASP GBD (residues 242–310), N-WASP_C_ (residues 193-501) and WASP GBD-VCA (residues 230–310 and 420–502 with a GGSGGS linker) constructs are described elsewhere [41]. For routine passage, all *E. coli* strains were grown in LB media at 37°C. Prior to infections, EHEC was cultured in DMEM+100 mM HEPES pH 7.4 in 5% CO_2_ to enhance type III secretion. HeLa, Cos7, and murine fibroblast-like cells (FLCs) [47] were used interchangeably and cultured in DMEM+10% FBS at 37°C in 5% CO_2_.

### Transfections and infections

All transfections were performed as described previously [31]. Infections for 3 h with non-pathogenic *E. coli* expressing intimin [31] and EHEC or EPEC strains [44]–[45] have also been described. To cluster Tir, cells were treated with antibodies that recognize its extracellular domain prior to the addition of formalin-fixed *S. aureus* Pansorbin particles (Calbiochem) [25].

### Immunofluorescence microscopy

Infected cells were fixed in 2.5% paraformaldehyde for 35 minutes and permeabilized with 0.1% Triton-X-100 in PBS as described previously [45]. Bacteria were identified with 1 µg/ml DAPI (Sigma), and F-actin was detected using 4 U/ml Alexa568-phalloidin (Molecular Probes). To visualize N-WASP derivatives, cells were treated with an anti-Flag M5 antibody (Sigma) and Alexa488 goat anti-mouse antibodies (Molecular Probes). To visualize HN-Tir derivatives, cells were treated with an HA.11 antibody (Covance) and Alexa488 or Alexa350 goat anti-mouse antibodies. To quantify the pedestal formation index in cells expressing high levels of N-WASP derivatives, which were identified by bright anti-Flag or GFP fluorescence, the percentage of cells harboring at least 10 adherent bacteria and 5 actin pedestals was measured. To quantify the pedestal index in cells infected with KC12, the percentage of cells harboring at least 10 adherent bacteria and 5 actin pedestals was measured. To quantify the pedestal index in cells treated with pansorbin particles, the percentage of HA-fluorescing cells harboring at least 10 adherent particles (the latter identified by virtue of their ability to bind fluorescently labeled secondary antibodies) and 5 actin pedestals was measured. At least 50 cells were examined per sample per experiment. Our previous work suggests that scoring of a pedestal index accurately reflects similar quantification methods that measure the fraction of bound bacteria that generate pedestals [25]. Cells expressing extremely high fluorescence levels of EspF_U_ were refractory to pedestal formation and were not included in these analyses. All scalebars are 1 µm in length.

### EspF_U_ interaction assays

For yeast two-hybrid assays. EspF_U_ variants consisting of different combinations of the 6 repeats were fused to the Gal4 transcriptional activation domain, whereas the N-WASP GBD was fused to the LexA DNA-binding domain. Pairwise combinations of these fusion proteins were tested for interactions by measuring activation of a *lacZ* reporter, as described [28]. For some pulldown assays (Figure 4B), cobalt-chelate conjugated magnetic Talon particles (Invitrogen) were saturated with His-EspF_U_-myc derivatives by incubating them with 75 µg/ml of each recombinant fragment for 1 h. After removal of unbound EspF_U_, beads were incubated with brain extract for 1 h in 50 mM NaPO_4_ pH 7.4, 150 mM NaCl, and 0.015% Triton X-100. Bound proteins were eluted by boiling in SDS-PAGE sample buffer. For other pulldown assays (Figure 4C), His-EspF_U_-myc derivatives were incubated in brain extract at specific concentrations for 0.5 h and collected using an excess of Talon particles. Bound proteins were again eluted by boiling in SDS-PAGE sample buffer.

### Immunoblotting

To prepare mammalian cell lysates, transfected cells were collected in PBS+2 mM EDTA and lysed in 50 mM Tris-HCl, pH 7.6, 50 mM NaCl, 1% Triton X-100, 1 mM Na_3_VO_4_, 1 mM PMSF, and 10 µg/ml each of aprotinin, leupeptin, pepstatin, and chymostatin (Sigma)), prior to mixing with SDS-PAGE sample buffer. Protein samples were boiled for 10 minutes, centrifuged, and analyzed by 10% SDS-PAGE prior to staining with Coomassie blue or transferring to nitrocellulose membranes and staining with Ponceau S. Membranes were blocked in PBS+5% milk (PBSM) before probing with N-WASP or GFP antibodies, as described previously [28]. Following washes, membranes were treated with secondary antibodies conjugated to alkaline phosphatase or horseradish peroxidase and developed using BCIP/NBT [45] or enhanced chemiluminescence [48].

### EspF_U_ expression and purification

His-EspF_U_-myc fusion proteins were expressed in *E. coli* BL21-Rosetta (Novagen) at 37°C in the presence of 0.1 mM IPTG for 3 h. Bacteria were lysed in 10 mM Tris pH 7.4, 150 mM NaCl, and protease inhibitor cocktail (Roche) using a cell disruptor (30kpsi, Constant Cell Systems). EspF_U_ was purified first using His-tag affinity for Ni-NTA-agarose beads (Qiagen) and eluted in lysis buffer containing 400 mM imidazole. To remove EspF_U_ degradation products, an anion exchange purification step, facilitated by the negatively charged 5myc-tag, was performed. EspF_U_ was bound to a HiTrap Q column (GE Healthcare) and eluted in 10 mM Tris pH 9.5 containing 1 M NaCl. Other EspF_U_ constructs (R′5, R′4-5, R1-5; Figure 8) with just an N-terminal His-tag were expressed in *E. coli* BL21-DE3 at 20°C in the presence of 1 mM IPTG for 16 h, and after Ni-NTA affinity purification the tag was cleaved with thrombin. Cleaved proteins were subjected to ion exchange and gel filtration chromatography to remove the tag and other impurities. Protein concentrations were estimated by Bradford assay (Bio-Rad).

### Preparation of brain extracts

Brains obtained from freshly slaughtered pigs (Dalehead Foods Ltd, Linton, UK) were cleaned, sectioned, and resuspended in extraction buffer (0.1 M MES pH 6.8, 1 mM EGTA, 0.5 mM MgCl_2_, 0.1 mM EDTA, 1 mM DTT, protease inhibitor cocktail), prior to homogenization using a Waring blender (2×15 s, 4°C). After clarification (7,000 g, 20 min, 4°C), extracts were filtered through cheesecloth and further clarified (11,000 g, 40 min, 4°C) prior to storage (−80°C). Extract to be subfractionated (60 ml) was dialysed against 2×5 L 20 mM Tris-Cl pH8, 2 mM MgCl_2_, 5 mM EGTA, 1 mM EDTA, 0.5 mM DTT, 0.2 mM ATP, 2.5% glycerol at 4°C, prior to loading onto tandem HiTrap Heparin columns (2×5 ml), pre-equilibrated with dialysis buffer. Bound proteins were eluted using a 0–0.5 M KCl gradient in dialysis buffer (2 ml/min) and 20 3 ml fractions collected. Fractions 11–17 contained both Arp2/3 complex and N-WASP upon immunoblotting, and were combined, dialysed (as above), concentrated 5-fold (Centricon), and stored at −80°C.

### N-WASP, WIP, and Arp2/3 expression and purification

His-tagged human N-WASP and WIP were expressed using recombinant baculovirus-infected High5 insect cells, as described in detail elsewhere (ADS and MDW, submitted). A recombinant N-WASP/WIP complex was purified by Nickel affinity chromatography followed by gel filtration chromatography to separate the complex from N-WASP or WIP alone. To maintain an autoinhibited N-WASP/WIP complex, freeze-thaw cycles and prolonged periods of storage on ice were kept to a minimum. Recombinant human Arp2/3 complex and native bovine Arp2/3 complex were purified as described previously [39],[41].

### Pyrene-actin assembly assays

Assays utilizing brain extract contained samples supplemented with 2.5 µM skeletal muscle actin (10% pyrene-labeled), and polymerization was measured as described previously [49]. Assays using N-WASP/WIP complex contained 2.0 µM actin (7% pyrene-labeled) and 20 nM recombinant Arp2/3 complex, and polymerization was measured as described previously [48]. Assays using the N-WASP GBD-VCA contained 4.0 µM actin (5% pyrene-labeled) and 10 nM bovine Arp2/3 complex, and polymerization was measured as described elsewhere [41].

### Isothermal titration calorimetry and gel filtration chromatography

Isothermal titration calorimetry was performed as described elsewhere [41]. Briefly, the WASP GBD was titrated into R′5, R′4-5, or R′1-5 in KMEI buffer (50 mM KCl, 1 mM MgCl_2_, 1 mM EGTA, and 10 mM imidazole pH 7.0) plus 5 mM β-mercaptoethanol. Prior to gel filtration analyses, the N-termini of EspF_U_ fragments R′5 and R′4-5 were fluorescently labeled by dialysis against 100 mM sodium bicarbonate pH 8.3 and treatment with a five-fold molar excess of AlexaFluor647-carboxylic acid, succinimidyl ester (Invitrogen) at 4°C for 16 hours. Extra dye was removed by desalting chromatography and subsequent dialysis. Conjugation was confirmed by mass spectrometry. Labeling efficiency was estimated as >97% by the ratio of absorbance at 280 and 650 nm. Interactions between Arp2/3 complex and N-WASP_C_ in complex with EspF_U_ were examined using a Superdex 200 10/300 GL column (GE Healthcare) equilibrated in KMEI plus 1 mM dithiothreitol buffer.
